# Carbon-Based Fiber Materials as Implantable Depth Neural Electrodes

**DOI:** 10.3389/fnins.2021.771980

**Published:** 2021-12-22

**Authors:** Xuefeng Fu, Gen Li, Yutao Niu, Jingcao Xu, Puxin Wang, Zhaoxiao Zhou, Ziming Ye, Xiaojun Liu, Zheng Xu, Ziqian Yang, Yongyi Zhang, Ting Lei, Baogui Zhang, Qingwen Li, Anyuan Cao, Tianzai Jiang, Xiaojie Duan

**Affiliations:** ^1^Department of Biomedical Engineering, College of Future Technology, Peking University, Beijing, China; ^2^School of Nano-Tech and Nano-Bionics, University of Science and Technology of China, Hefei, China; ^3^Key Laboratory of Multifunctional Nanomaterials and Smart Systems, Advanced Materials Division, Suzhou Institute of Nano-Tech and Nano-Bionics, Chinese Academy of Sciences (CAS), Suzhou, China; ^4^School of Materials Science and Engineering, Peking University, Beijing, China; ^5^Academy for Advanced Interdisciplinary Studies, Peking University, Beijing, China; ^6^Brainnetome Center, Institute of Automation, Chinese Academy of Sciences (CAS), Beijing, China; ^7^National Biomedical Imaging Center, Peking University, Beijing, China

**Keywords:** brain activity mapping, multi-modal neural interfacing, soft bioelectronics, carbon nanomaterials, biocompatibility

## Abstract

Implantable brain electrophysiology electrodes are valuable tools in both fundamental and applied neuroscience due to their ability to record neural activity with high spatiotemporal resolution from shallow and deep brain regions. Their use has been hindered, however, by the challenges in achieving chronically stable operations. Furthermore, implantable depth neural electrodes can only carry out limited data sampling within predefined anatomical regions, making it challenging to perform large-area brain mapping. Minimizing inflammatory responses and associated gliosis formation, and improving the durability and stability of the electrode insulation layers are critical to achieve long-term stable neural recording and stimulation. Combining electrophysiological measurements with simultaneous whole-brain imaging techniques, such as magnetic resonance imaging (MRI), provides a useful solution to alleviate the challenge in scalability of implantable depth electrodes. In recent years, various carbon-based materials have been used to fabricate flexible neural depth electrodes with reduced inflammatory responses and MRI-compatible electrodes, which allows structural and functional MRI mapping of the whole brain without obstructing any brain regions around the electrodes. Here, we conducted a systematic comparative evaluation on the electrochemical properties, mechanical properties, and MRI compatibility of different kinds of carbon-based fiber materials, including carbon nanotube fibers, graphene fibers, and carbon fibers. We also developed a strategy to improve the stability of the electrode insulation without sacrificing the flexibility of the implantable depth electrodes by sandwiching an inorganic barrier layer inside the polymer insulation film. These studies provide us with important insights into choosing the most suitable materials for next-generation implantable depth electrodes with unique capabilities for applications in both fundamental and translational neuroscience research.

## Introduction

Implantable depth neural electrodes constitute the basis for a wide range of applications, including deciphering how information is encoded inside the brain (Buzsáki, 2004; Stanley, 2013), treating various neurological diseases (Borchers et al., 2012; Lozano and Lipsman, 2013; Cash and Hochberg, 2015), and realizing brain-machine interfaces (BMIs) (Bensmaia and Miller, 2014; Shenoy and Carmena, 2014; Choi et al., 2016; Lebedev and Nicolelis, 2017). The capability of spatiotemporal mapping at the single-neuron level is advantageous over electroencephalography (EEG) or electrocorticography (ECoG) surface probes (Kim et al., 2010; Buzsáki et al., 2012), or non-invasive brain imaging methods such as functional magnetic resonance imaging (fMRI) (Logothetis et al., 2001; Weiskopf et al., 2007; Gosselin et al., 2011; Figee et al., 2013) or functional near infrared spectroscopy (fNIR) (Izzetoglu et al., 2005; Bunce et al., 2006; Ayaz et al., 2007, 2009; Harrison et al., 2014). Despite this advantage, single neuronal recordings with implantable depth electrodes remain limited in several aspects, including the limited number of sampling sites and challenges in achieving chronically stable operation (Vitale et al., 2015). Combining electrophysiological measurements with simultaneous whole-brain imaging techniques, such as fMRI, provides a useful solution to alleviate the challenge in scalability of implantable depth electrodes (Zhao et al., 2020). However, many commonly used metals for implantable depth electrodes elicit significant magnetic resonance imaging (MRI) artifact due to the mismatch in magnetic susceptibility between metal and water/tissues, which obstructs functional and structural mapping of a large volume of brain tissues surrounding the electrodes (Zhao et al., 2016). Implantable depth electrodes with high MRI compatibility are important for combining high-resolution electrophysiological measurements with more global MRI mapping of brain activity for fundamental neuroscience studies, as well as clinical evaluation and monitoring.

Under mechanical mismatch between the implantable electrodes and brain tissue, the natural micromotion of the host brain tissue induces intense stress at the electrode-brain interface. This stress causes repetitive mechanical stimulation and injury on tissues and results in sustained inflammatory responses, leading to neuronal loss and glial scar formation around the electrodes (Williams et al., 1999; Seymour and Kipke, 2007; Zhong and Bellamkonda, 2008; Jeong et al., 2015). Minimizing the chronic inflammatory tissue responses of the electrodes is thus critical to achieve chronically stable neural recording and stimulation. Recent research has shown that increasing the mechanical compliance of the implantable electrodes can effectively alleviate the chronic inflammatory responses and reduce gliosis formation (Xie et al., 2015; Fu et al., 2016; Liu, 2018; Lu et al., 2019). The flexibility of the implantable depth electrodes, characterized by bending stiffness *K* which is the ratio between the longitudinal loading force and the displacement, is strongly dependent on the electrode size. Materials with excellent interfacial electrochemical properties, including low electrode impedance and high charge injection capability, are thus highly desirable for flexible electrodes fabrication because they allow for further electrode miniaturization while maintaining reasonable electrode electrochemical performance.

Structural failures of neural electrodes upon implantation constitute another prevalent failure mode of neural recording and stimulation, in addition to neuronal degeneration and glial scar encapsulation around the implanted electrodes. The stress at the electrode–tissue interface can accelerate the material degradation which results in cracking, blistering, and delamination of the electrode insulation layers, eventually contributing to the failure of neural recording and stimulation (Prasad et al., 2012, 2014; Gilgunn et al., 2013; Kozai et al., 2015). Thin polymer films are commonly used insulation layers for implantable depth electrodes due to their softness, good biocompatibility, and readiness for conformal coating. However, these polymer encapsulation layers showed intrinsic limitations in water permeability and extrinsic effects associated with localized defects (arising from the growth process, i.e., pinholes, cracks, and grain boundaries) (Li et al., 2010, 2019; Minnikanti et al., 2014), which may strongly affect the dielectric properties of the material and lead to electrical leakage under long-term degradation in physiological conditions. Increasing the thickness of the electrode insulation layer is an effective way to improve its durability and stability under physiological environment. However, the thickness increase of the insulation layer is accompanied by a significant reduction of the mechanical compliance of the electrodes. Exploring strategies of improving the durability and stability of the insulation under physiological conditions without sacrificing the mechanical compliance of the electrodes is critical to achieve long-term, stable chronic neural recording and stimulation at high spatiotemporal resolution.

Carbon-based materials have emerged as new candidates for implantable neural interfaces. Carbon fibers (CFs) were used to fabricate electrodes with the assistance of poly (3,4-ethylenedioxythiophene) (PEDOT) coating, which enabled single-neuron recording in acute and early chronic experiments in rats (Kozai et al., 2012; Patel et al., 2016). Electrodes made from carbon nanotube fibers (CNTFs) were demonstrated to be capable of continuously detecting and isolating single neuronal units from rats in early chronic scale (Vitale et al., 2015) and for up to 4–5 months (Lu et al., 2019) without electrode repositioning, with greatly reduced brain inflammatory responses as compared to their stiff metal counterparts. Graphene fibers (GFs) electrodes were found to have a charge-injection-limit (CIL) of ∼10 mC/cm^2^, which is higher than most commonly used electrode materials for neural stimulation (Apollo et al., 2015; Zhao et al., 2020), and capable of detecting neuronal activity (Apollo et al., 2015) with a high signal-to-noise ratio (SNR) of 9.2 dB (Wang et al., 2019). The CFs were also used extensively in electrochemical neurotransmitter detection both *in vitro* and *in vivo* (Huffman and Venton, 2009; Özel et al., 2011; Khan et al., 2014; Durairaj et al., 2018). In addition, electrodes made of carbon-based fibers showed greatly reduced MRI artifacts compared with electrodes made of metals such as PtIr (Lu et al., 2019; Zhao et al., 2020). These results demonstrated the advantages of carbon-based fibers in building multi-modal and chronically stable neural interfaces. In this work, we aim to provide a systematic comparative evaluation on the electrochemical properties, mechanical properties, and MRI compatibility of different kinds of carbon-based fiber materials, and to explore solutions to improve the durability and stability of the insulation layer without sacrificing the flexibility of the implantable depth electrodes. These studies will provide us with important insights into choosing the most suitable materials for next-generation implantable depth electrodes with unique capabilities for applications in both fundamental and applied neuroscience research.

## Materials and Methods

### Electrode Fabrication

In this work, we fabricated neural depth implantable electrodes from four types of carbon-based fiber materials, including two types of CNTFs, one type of GFs, and one type of CFs. Array CNT fibers (aCNTFs) were dry-drawn from a vertically super-aligned array of CNTs grown by chemical vapor deposition (CVD) on a silicon substrate (Jia et al., 2011) (see Supplementary Methods for details). The second type of CNTFs are floating catalyst CNT fibers (fCNTFs), which were spun from the CNT membrane grown with a floating catalyst CVD method using a liquid source of carbon and an iron nanocatalyst (Zhou et al., 2021) (see Supplementary Methods for details). Compared to aCNTFs, fCNTFs have higher content of iron contamination and also higher electrical conductivity (Bulmer et al., 2021). The aCNTFs and fCNTFs used in our work had twisted angles of 25° and 10°, respectively. The GFs were prepared through a dimension-confined hydrothermal process from aqueous graphene oxide (GO) suspensions (Dong et al., 2012). This gave GFs with excellent electrical conductivity and mechanical robustness. Market available CFs (C3005, World Precision Instruments, United States) were used to fabricate the CF electrodes. All these carbon-based fibers had a diameter of 30 μm unless specified otherwise.

For electrode fabrication, the fibers were first coated with Parylene-C film of 3–5 μm thickness in a Parylene coater (PDS 2010 Labcoter, Specialty Coating Systems, United States). The thickness of the Parylene-C coating layers was confirmed *via* scanning electron microscopy (SEM). One end of the insulated fibers was soldered onto a printed circuit board or a metal pin connector used to connect to the external electronics. The soldering points were sealed and stabilized with a thin layer of epoxy. The active sites of the electrodes were exposed by blade cut. The same method described above was used to fabricate the platinum (Pt) electrodes from Pt microwires (30 μm diameter, XYφ0.03, XIYU Mechanical and Electrical Technology Co., China) for a comparison.

### Electrode Characterization

An electrochemical workstation (CHI660e, CH Instruments, United States) was used to perform all electrochemical characterization. Impedance spectroscopy (EIS) and cyclic voltammetry (CV) were measured in 1x phosphate-buffered saline (PBS, pH 7.4) at room temperature in a three-electrode cell comprising an Ag| AgCl served as the reference electrode, a large area platinum foil (50 mm × 50 mm, 0.15 mm thickness, GF29782312, Merk, Germany) as the counter electrode, and the tested electrode as the working electrode. EIS was performed in the range of 10∼100 kHz. CV was scanned between potentials of −0.6 and 0.8 V at a rate of 50 mV/s, beginning at open-circuit potential and sweeping in the positive direction first. Each sample was swept for two cycles. The cathodic charge-storage-capacity (CSCc) was calculated as the time integral of the cathodic current recorded in the second cycle. For water window testing, CV was performed at a scan rate of 1 mV/s. The water window was determined as the water oxidation and reduction potential obtained from CV measurements, where a steep increase in current was observed (Supplementary Figure 1).

For CIL measurement using voltage transient experiments, a three-electrode cell (the same as above) was used. Biphasic, symmetric, and charge-balanced square-wave current pulses of 60 μs duration were delivered to the tested samples at a frequency of 130 Hz with a stimulator (Model 2100, A-M Systems, United States). Voltage transients under the current pulses were recorded with an oscilloscope (DSO5202P, Hantek, China), and the negative potential excursion was calculated by subtracting the initial access voltage due to solution resistance from the total voltage (Zhao et al., 2020). The CIL was calculated by multiplying the current amplitude and pulse duration at which the negative potential excursion reaches the water reduction limit (−1.5 and −0.6 V for carbon-based fiber electrodes and Pt electrodes, respectively), divided by the geometric surface area (cross-sectional area) of the electrodes. All the electrodes used in the electrochemical characterization were made from carbon-based fibers or Pt microwires insulated with ∼5 μm thick Parylene-C film.

The Young’s modulus and tensile strength of each type of material were measured from the stress–strain curves on a single-column testing instrument (Instron 5843, Instron Corp., United States) (Supplementary Figure 2). The bending stiffness *K*, which is the ratio between the longitudinal loading force and the displacement, represents the mechanical characteristics of the tissue–electrode interface. For a cylindrical beam, *K* can be estimated as (Steif, 2012):


$$K=E\,\pi \,\frac{d^{4}}{64}$$

where *E* is Young’s modulus of the material and *d* is the diameter of the beam.

### Insulation Stability Improvement and Test

We explored the strategy of using alternating polymer/inorganic multilayers for insulation to improve the long-term stability of the carbon-based fiber electrodes. The aCNTFs with a diameter of 15 μm were first coated with 2 μm thick Parylene-C (PDS 2010 Labcoter, Specialty Coating Systems, United States). Then atomic layer deposition (ALD) of 10 nm HfO_2_/20 nm Al_2_O_3_/10 nm HfO_2_ triple layer was carried out at 100°C at 0.1 nm/min using the ALD System (Savannah S200, Ultratech, United States). Finally, another 2-μm-thick Parylene-C layer was coated to finish the insulation. These electrodes are labeled as “Test 1” samples. “Test 2” samples were prepared same way except for that for each layer of Parylene-C, 1 μm thickness was used. Electrodes insulated with 4 and 2 μm Parylene-C were labeled as “Control 1” and “Control 2” samples, respectively, and used for comparison of the electrode stability. “Test 1” and “Control 1,” “Test 2” and “Control 2” samples were placed side by side, respectively, for Parylene-C deposition to avoid the variance in film thickness and quality from batch to batch.

Accelerated aging test (AAT) was performed to test the stability of the above aCNTF electrodes. The electrodes were completely submerged in 1x PBS at 60°C in an electro-thermostatic water bath (CU-420, Shanghai Yiheng Instrument Co., China) throughout the test. The use of 60°C instead of the physiological temperature of 37°C was to accelerate the degradation of the electrode coatings. ASTM F1980 (American Society for Testing and Materials Standard guide for accelerated aging of sterile medical device packages) recommends that aging temperature do not exceed 60°C to avoid non-linear variations in the rate of reaction (Bierwagen et al., 2003), therefore we maintained a constant 60°C during our experiments. Temperature variations were below ± 0.5°C throughout the experiment. PBS solutions in the vials was replaced every day to keep a stable concentration and pH value. The electro-thermostatic water bath was sealed to avoid the solution evaporation. The aCNTF electrodes was soldered onto a single-row, multi-pins straight male headers tip which was bridged on the opening of the vial. This way the electrodes were immersed in PBS while the connectors were kept out of the solution.

We used the following formula and assumptions to extrapolate the simulated age at body temperature (Age_37°*C*_) from that at the AAT temperature (Age_60°*C*_) (Hukins et al., 2008):


A⁢g⁢e37⁢C°=(A⁢g⁢e60⁢C°)×Q10(TA⁢A-TR⁢S)/10

Where Q_10_ = 2 (a 10°C increase in temperature doubles rate of the chemical reaction), T_*AA*_ = 60°C (accelerated aging temperature) and T_*RS*_ = 37°C (recommended shelf temperature-body temperature). From this calculation, the age of 153 days at 60°C corresponds to an age of 753 days at body temperature.

### Magnetic Resonance Imaging Compatibility Studies

All procedures for handling the animals were approved by the Institutional Animal Care and Use Committees of Peking University (#COE-DuanXJ-1). All MRI experiments were performed using a 9.4 T animal MRI scanner (Bruker BioSpin 94/20USR MRI, Germany) with Bruker’s 86 mm volume coil for transmission and a four-channel rat head surface coil for receiving (ParaVision Version 6.0.1 for MRI acquisitions). The electrodes made from carbon-based fibers or Pt wires coated with ∼5 μm Parylene-C were implanted in rat brains as described previously (Lu et al., 2019). For each animal used, all the five types of electrodes were implanted. A total of five rats were used for the MRI study. The connectors were not included in the electrodes to avoid their influence on MRI. The electrodes were tethered and cemented to the skull. Two craniotomies (∼2 × 3 mm^2^ each) were performed directly above two areas of interest (AP: −2.0∼−4.0 mm; ML: ± 1.0∼3.0 mm; DV: −4.0∼−6.0 mm from dura). The five types of electrodes were implanted in these areas with a ∼0.8 mm distance between the adjacent electrodes, with positions shown as in Figure 5. Craniotomies were sealed with a silicone elastomer (Kwik-Sil, World Precision Instruments, United States). MRI scans were conducted 1–3 days after the electrode implantation surgery. After anesthesia with 4% isoflurane, the animal’s head was fixed in the MRI scanning coil with the body axis at the centerline to perform T_2_- and echo-planar imaging (EPI)-weighted horizontal and coronal plane scans following the calibration scan. During MRI scanning, 1.5% isoflurane with 100% medical air delivered *via* a nose cone was used to maintain anesthesia. Animal temperature, respiration, and blood oxygen saturation were all monitored and within normal ranges (Model 1025, SA Instruments, United States). Body temperature was maintained at 37 ± 0.5°C using a circulated hot water bed and a hot air blower.

**FIGURE 1:**
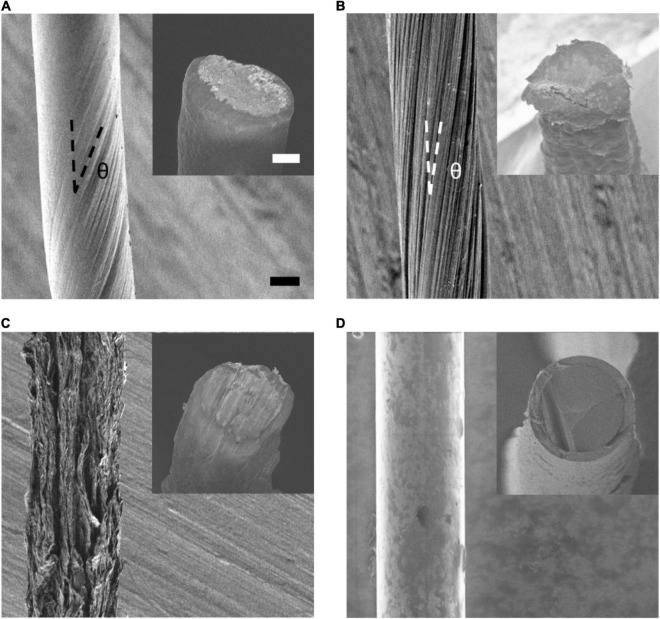
SEM characterization. **(A–D)**, SEM images of the side view of an aCNTF **(A)**, fCNTF **(B)**, GF **(C)**, and CF **(D)**. The twisted angle is marked as θ. Insets of panels **(A–D)**, SEM images of the tip of an aCNTF, fCNTF, GF, and CF electrode. Scale bars of 10 μm apply to all panels and insets.

**FIGURE 2:**
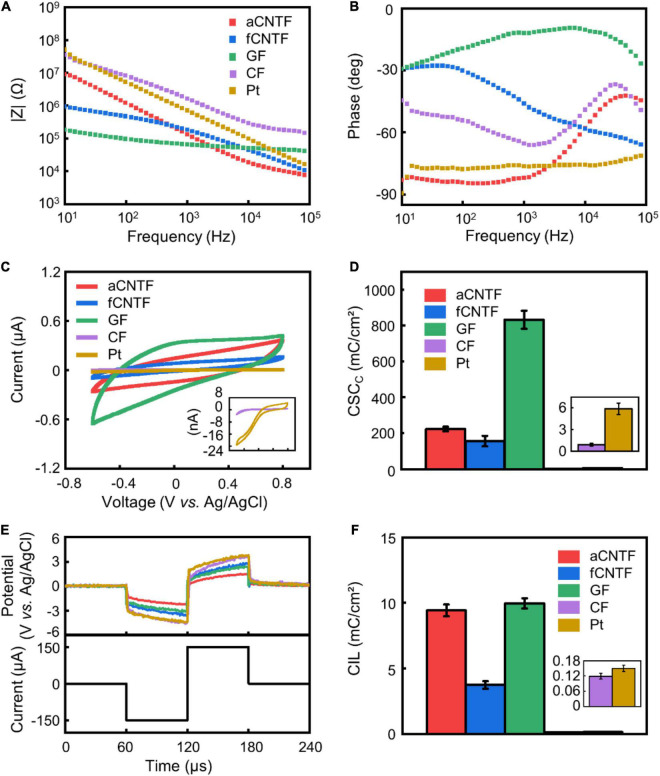
Electrochemical properties. **(A–C)** representative impedance magnitude **(A)**, phase **(B)**, and CV **(C)** of various electrodes. **(D)** Calculated CSC_*c*_. **(E)** Representative voltage transient of various electrodes (upper curves) in response to a current pulse of 150 μA amplitude (lower curve). **(F)** CIL of different electrodes. All electrodes were made of carbon-based fibers or Pt wires with 30 μm diameter insulated with 5 μm thick Parylene-C. The same color codes in **(A)** are used in **(B,E)**. Error bars in **(D,F)** show SEM (*n* = 8 for **D** and *n* = 5 for **F**).

**FIGURE 3:**
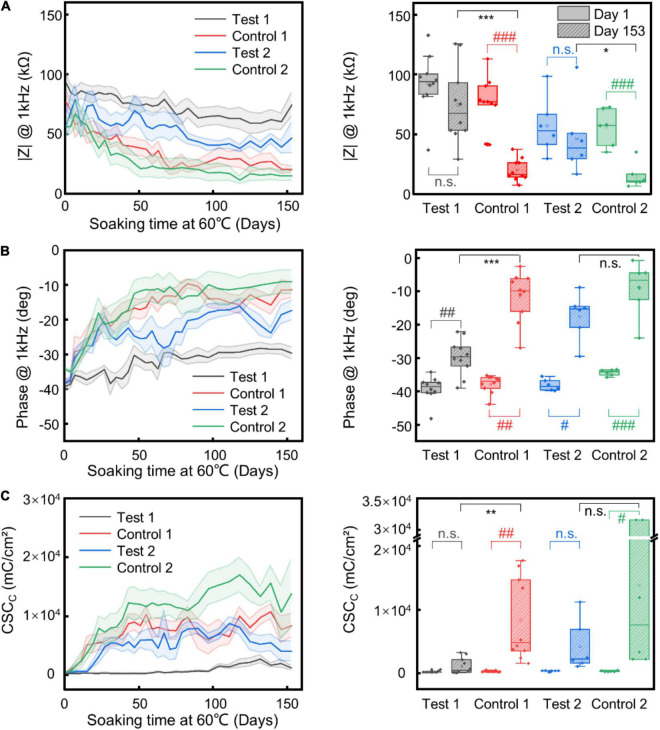
Insulation stability test. Change and statistical comparison analysis of impedance magnitude **(A)** and phase **(B)** at 1 kHz, and CSC_*c*_ **(C)** of different electrode samples upon time under AAT. The shaded regions represent the SEM (*n* = 10 for “Test 1” and “Control 1” electrodes, *n* = 6 for “Test 2” and “Control 2” electrodes). For all comparisons, Shapiro–Wilk was used to test normality and Brown-Forsythe was used to test the homogeneity of variance. For comparison between Day 1 and Day 153 of the same type of insulation layer, paired sample *T*-test or paired Wilcoxon signed-rank test (when test for normality or equal variance failed) was used. For comparison between test and control group on Day 153, two sample *T*-test or Mann–Whitney test (when test for normality or equal variance failed) was used. * corresponds to comparison between test and control group on Day 153; # corresponds to comparison between Day 1 and Day 153 of the same samples. *, #, *p* ≤ 0.05; **, ##, *p* ≤ 0.01; ***, ###, *p* ≤ 0.001.

**FIGURE 4:**
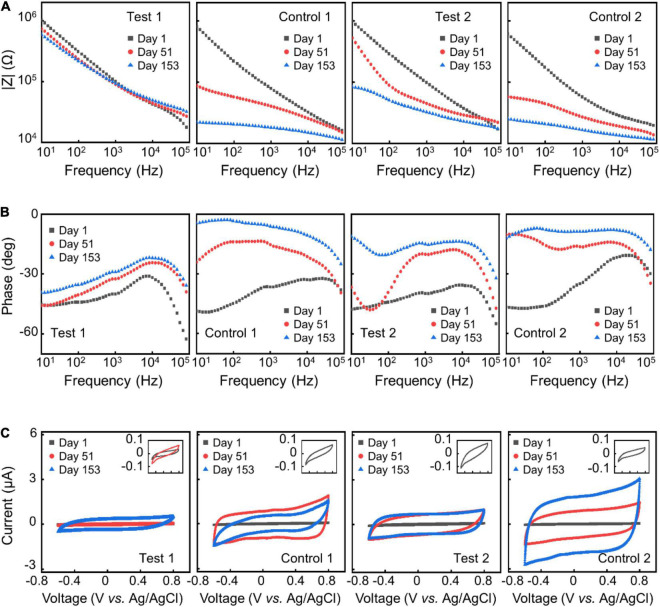
AAT results. Representative impedance spectra **(A,B)** and CV **(C)** of different electrode samples at day 1, day 51, and day 153 in ATT.

**FIGURE 5:**
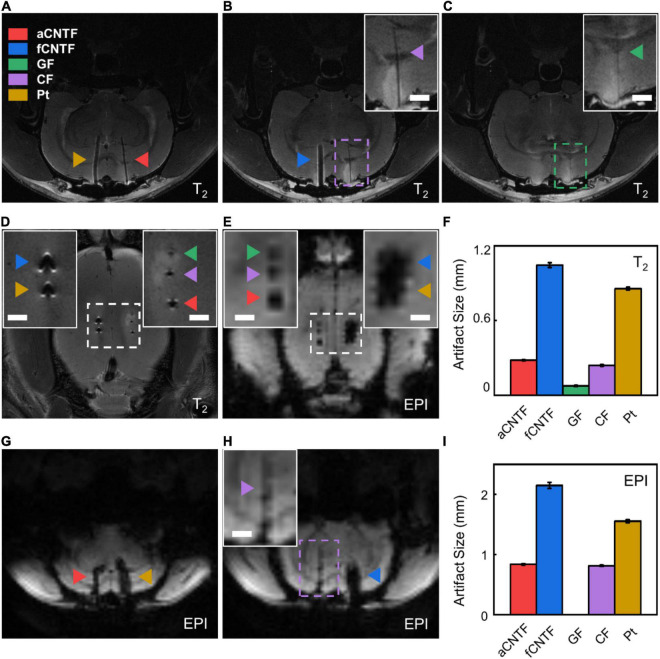
*In vivo* MRI artifact assessment. **(A–C)** Coronal sections of the T_2_-weighted images of a rat implanted with various electrodes. **(D,E)** Horizontal sections of T_2_-weighted **(D)** and EPI **(E)** images of a rat implanted with various electrodes. **(F)** T_2_ artifact size of different electrodes. **(G,H)** Coronal sections of the EPI-weighted images of a rat implanted with various electrodes. **(I)** EPI artifact size of different electrodes. All electrodes were made from carbon-based fibers or Pt wires of 30 μm diameter insulated with ∼5 μm thick Parylene-C. The insets are zoomed-in photographs of the dashed boxes. Scale bar, 1 mm. Error bars in **(F,I)** show SEM. For each type of electrode, *n* = 5 from five animals.

T_2_-weighted anatomical images were acquired with parameters as follows: TR/TE = 2200/33 ms, RARE factor = 8, field-of-view (FOV) = 28 × 28 mm^2^, matrix size = 512 × 512, and slice thickness = 0.6 mm. EPI-weighted images were acquired with parameters as follows: TR/TE = 500/13 ms, FOV = 30 × 30 mm^2^, matrix size = 80 × 80, flip angle = 55°, slice thickness = 0.6 mm, and segment = 4. To quantify the MRI artifact size of the electrodes, raw coronal images with the largest electrode artifact were selected. EPI images were upsampled from 0.37 × 0.37 × 0.6 mm^3^ to 0.05 × 0.05 × 0.6 mm^3^ voxel resolution. No upsampling was done for T_2_ images. The edges of artifacts were detected with Canny Edge Detector using Matlab (R2018b, Mathworks, United States). The artifact size in the medial-lateral direction was then calculated from the pixel numbers and averaged over different animal subjects (Supplementary Figure 3).

## Results and Discussion

### Comparison on Electrochemical Performance

Different carbon-based fibers show distinct surface morphology due to their different microstructure. As shown in Figure 1, side view of SEM images of aCNTFs and fCNTFs shows aligned CNT bundles twisted around the fiber axis at characteristic angles. While for the GFs, the SEM image shows a porous structure with rough surface. CFs showed a much smoother surface morphology than that of the CNTFs and GFs. The SEM images of the exposed cross sections (inset of Figure 1) of various electrodes showed a rough and porous microstructure for aCNTF, fCNTF, and GF electrodes, which is important to achieve a large specific surface area, thus helpful to obtain better electrochemical performance for neural electrode application.

The magnitude of impedance at 1 kHz is a commonly used metric for neural electrodes. Figures 2A,B show representative EIS of various electrodes. EIS measurements gave similar impedance magnitude of 129.49 ± 5.19 kΩ and 162.56 ± 20.06 kΩ (mean ± SEM, *n* = 8. Same for below) at 1 kHz for electrodes made from the aCNTFs and fCNTFs. This value was lower than that of Pt electrodes (536.58 ± 55.53 kΩ at 1 kHz), consistent with previous results that the CNTFs have superior interfacial electrochemical properties than Pt or PtIr wires (Vitale et al., 2015; Ganji et al., 2017; Lu et al., 2019; Zhao et al., 2020). GF electrodes showed the lowest impedance value of 50.44 ± 5.21 kΩ at 1 kHz. And CF electrodes showed impedance values of 2.11 ± 0.40 MΩ at 1 kHz, higher than all other electrodes. Compared to aCNTF and CF electrodes, the phase of the fCNTF electrodes showed a less capacitive characteristics. This may be related with the high iron content of the fCNTFs which provided extra redox sites. The GF electrodes showed largest CSC_*c*_ of 832.03 ± 50.18 mC/cm^2^ (mean ± SEM, *n* = 8. Same for below) among all tested electrodes (Figures 2C,D). The aCNTF and fCNTF electrodes exhibited slightly lower CSC_*c*_ of 223.86 ± 12.78 and 156.21 ± 29.37 mC/cm^2^, respectively. And the Pt and CF electrodes showed lower CSC_*c*_ of 5.88 ± 0.78 and 0.91 ± 0.19 mC/cm^2^, respectively.

Voltage transient measurements were carried out to estimate the CIL, which is defined as the maximum charge that can be injected in a current-controlled stimulation pulse without polarizing an electrode beyond the potentials for water reduction or oxidation (Cogan, 2008; Zhao et al., 2020). Compared to Pt electrodes, the carbon-based fiber electrodes showed larger water window (Supplementary Figure 1). Based on the water window measurement, we calculated the CIL using −1.5 and −0.6 V as the water reduction limit for carbon-based fiber electrodes and Pt electrodes, respectively. The aCNTF and GF electrodes showed comparable CIL of 9.43 ± 0.45 mC/cm^2^ (mean ± SEM, *n* = 5. Same for below) and 9.96 ± 0.39 mC/cm^2^ (Figures 2E,F) respectively, which is slightly higher than that of the fCNTF electrodes (3.74 ± 0.29 mC/cm^2^). The CF and Pt electrodes exhibited much lower CIL of 0.12 ± 0.01 and 0.15 ± 0.01 mC/cm^2^, respectively. A comparison of our results with literature was included as Supplementary Table 1.

The low CIL and CSC_*c*_ indicate that when using CF or Pt electrodes to inject charge safely for neural stimulation, a much larger electrode size is required. The significantly larger impedance and lower charge injection capability indicate poor interfacial electrochemical property of the CFs, consistent with previous reports (Kozai et al., 2012; Pancrazio et al., 2017). The GF, aCNTF, and fCNTF microelectrodes showed excellent electrochemical performance which arises from the high surface area of these fibers accessible to ions due to the interstitial spaces between the aligned CNTs, CNT bundles, and GO sheets constituting the fibers (Dong et al., 2012; Lu et al., 2019). These superior interfacial electrochemical properties of GF, aCNTF, and fCNTF electrodes allow a much smaller size to be used, which can effectively increase the mechanical compliance of the electrodes, thus reducing the inflammatory responses and helping to achieve long-term stable neural recording and stimulation.

### Comparison on Mechanical Properties

Table 1 compares the mechanical characteristics of each type of material. The aCNTFs, fCNTFs, and GFs are more mechanically compliant with smaller Young’s modulus compared to CFs and Pt wires. The bending stiffness *K* scales with material rigidity (Young’s modulus) *E* linearly but scales with the beam diameter *d* to the fourth power. The improved electrochemical properties of the aCNTFs, fCNTFs, and GFs permit a much smaller diameter to be used. This can drastically reduce the bending stiffness of electrodes, thus mitigating mechanical stress at the electrode–tissue interface and reducing the inflammatory responses (Muthuswamy et al., 2011; Lu et al., 2019). Compared to other carbon-based fibers, the GFs showed lower tensile strength. A high tensile strength helps to achieve a high success rate in electrode fabrication, handling, transportation, and implantation. The low tensile strength of the GFs makes them less mechanically strong especially when the diameter is decreased. The CNTFs, with combined improved electrochemical performance, mechanical compliance, and tensile strength, are ideal candidates for fabricating flexible implantable depth electrodes.

**TABLE 1:** Mechanical properties of different materials.

Material	Twisted angle (°)	Young’s modulus (GPa)	Tensile strength (MPa)	Bending stiffness *K* (nN.m^2^)
aCNTF	25	10.89 ± 1.22	741.21 ± 46.05	0.43
fCNTF	10	22.26 ± 0.61	925.55 ± 23.35	0.88
GF	–	12.49 ± 1.04	168.39 ± 18.78	0.50
CF	–	51.86 ± 1.86	899.28 ± 39.93	2.06
Pt		81.72 ± 4.43	622.22 ± 13.95	3.25

*Error bars show SEM (n = 6).*

### Stability Improvement

To improve the structural durability and stability of the neural electrodes without sacrificing the flexibility of the electrodes, we explored the strategy of using alternating stacks of polymer/inorganic materials as insulation layers. We carried out AAT to characterize the structural and functional stability of different insulation strategies. Figure 3 shows the change of impedance amplitude and phase at 1 kHz, and CSC_*c*_ upon time from the AAT, with some representative impedance spectra and CV curves shown in Figure 4. The impedance amplitude at 1 kHz of “Test 1” samples decreased by less than 20% after 153 days of AAT. Nevertheless, “Control 1” samples which have the same thickness of Paylene-C as “Test 1” samples but with no sandwiching inorganic layer in the encapsulation exhibited a large drop of over 70% in impedance amplitude at 1 kHz after 153 days of AAT. Meanwhile, the impedance phase of “Test 1” samples exhibited a slight increase while that of “Control 1” samples exhibited a significant change from −38° to −11° after 153 days of AAT. The CSC_*c*_ of the “Test-1” samples showed no statistically significant difference from that on Day 1, distinct from that of the “Control 1” samples which showed a ∼26-fold increase. The significant decrease in impedance amplitude, increase in impedance phase and CSC_*c*_ of “Control 1” samples indicates a leakage in the insulation layer of the electrodes. The “Test 2” samples also showed significant improvement in stability compared to “Control 2” samples, manifested as smaller change in impedance amplitude, impedance phase, and CSC_*c*_. By extrapolating the simulated age at body temperature from the age at the AAT temperature, we conclude that by using stacks of polymer/organic layers for electrode insulation, the aCNTF electrodes could be stable at physiological conditions at least for up to 753 days.

These results indicated that by sandwiching an inorganic HfO_2_/Al_2_O_3_/HfO_2_ ALD film inside the Parylene-C layer as a barrier layer, the durability and stability of the insulation can be significantly improved. Inorganic materials including Al_2_O_3_, SiN_*x*_, etc., which are grown directly by techniques such as ALD and CVD, offer superior insulation properties (Minnikanti et al., 2014; Ahn et al., 2019; Li et al., 2019). Nevertheless, in most cases, especially those encountered in research laboratories, these films are often suffered from extrinsic limitations associated with heterogeneities in the growth processes and/or contaminants which leads to micro/nanoscale material defects including pinholes, cracks, nanopores, and grain boundaries, etc. (Li et al., 2010, 2019; Minnikanti et al., 2014). By sandwiching an inorganic barrier layer inside the polymer encapsulation, the potential of such types of defects to extend throughout the whole thickness of the insulation layer can be effectively reduced, thus improving the durability and stability of the insulation. The Al_2_O_3_ film from ALD is reported to possess low water permeability (Xie et al., 2012), but easily dissolves through hydrolysis when contacting with aqueous solutions (Abdulagatov et al., 2011; Correa et al., 2015; Nehm et al., 2015; Daubert et al., 2017; Kim et al., 2017). On the other hand, HfO_2_ is chemically inert and insoluble in aqueous solutions which makes it suitable as a capping layer of Al_2_O_3_ to increase its barrier performance in a liquid water environment (Kim et al., 2017; Ahn et al., 2019). The use of the nanometer thick inorganic layers from ALD doesn’t add too much in electrode diameter, thus will not compromise the mechanical compliance of the electrodes. Although the final validation of this insulation strategy will require chronic implantation and *in vivo* neural recording from large number of animal models, we think our work here provides a possible way to achieve neural recording and stimulation at high spatiotemporal resolution over time scales spanning the lifetime of many animal models for biomedical research. In addition, we believe that the strategy of using alternating stacks of polymer/inorganic materials as insulation layers could work for other types of neural electrodes, including metal microwire electrodes, silicon probes, and thin film electrode array too.

### Comparison on Magnetic Resonance Imaging Compatibility

We compared the MRI image artifacts of different electrodes implanted into rat brains in a high-field 9.4T MRI scanner. All electrodes were made from carbon-based fibers or Pt microwires insulated with 5-μm-thick Parylene-C. As shown in Figure 5, GF electrodes showed smallest T_2_ artifacts of 0.075 ± 0.005 mm (mean ± SEM, *n* = 5. Same for below) and were undetectable in EPI coronal images. The aCNTF and CF electrodes showed comparable artifact sizes of 0.281 ± 0.004 mm and 0.240 ± 0.006 mm, respectively, for T_2_ sequence and 0.838 ± 0.012 mm and 0.814 ± 0.012 mm, respectively, for EPI sequence. The fCNTF electrodes elicited largest artifact with 1.045 ± 0.019 mm T_2_ artifact and 2.150 ± 0.049 mm EPI artifact. This is consistent with the fact that fCNTFs have high content of iron contamination from CNTs growth process (Zhou et al., 2021). The artifact size of the Pt electrodes was 0.856 ± 0.011 mm for T_2_ sequence and 1.556 ± 0.026 mm for EPI sequence, which is slightly smaller than those from fCNTF electrodes and much larger than other carbon-based electrodes.

The results indicate that GFs are the most suitable material for fabricating highly MRI-compatible neural electrodes. But the relatively low tensile strength limited their miniaturization for flexible electrodes fabrication. The aCNTF and CF electrodes could be alternative choices for MRI-compatible electrodes. However, due to their poor interfacial electrochemical performance as described above, a much larger diameter will have to be used when using CF electrodes for neural recording and stimulation. This will further increase the MRI artifact size and makes them less desirable for MRI-compatible neural electrodes. The aCNTF electrodes have excellent electrochemical and mechanical properties, which permits the fabrication of small diameter neural electrodes. Together with their reasonably small MRI artifact, the aCNTF will be a suitable choice for fabricating flexible and MRI-compatible neural electrodes for stable longitudinal studies involving simultaneous electrophysiological measurements and anatomical or functional MRI.

## Conclusion

Our study here demonstrated that the nanocarbon-based fibers, including aCNTFs, fCNTFs, and GFs, have superior electrochemical performance for neural electrodes fabrication. The low Young’s modulus and high tensile strength of aCNTFs and fCNTFs, combined with their superior electrochemical performances, made them especially suitable for fabricating implantable depth electrodes with small size and high flexibility, which is expected to be associated with reduced inflammatory responses. Combining the strategy of sandwiching inorganic barrier layers using ALD inside the polymer insulation to improve the structural stability will provide us a promising way to achieve long-term stable neural recording and stimulation spanning the lifetime of the animal models for biomedical research. The GF electrodes showed highest MRI compatibility. But the aCNTFs are more suitable for fabricating flexible and MRI-compatible electrodes due to their excellent electrochemical performance, mechanical compliance, and strength, as well as high MRI compatibility.

## Data Availability Statement

The raw data supporting the conclusions of this article will be made available by the authors, without undue reservation.

## Ethics Statement

The animal study was reviewed and approved by the Institutional Animal Care and Use Committees of Peking University (#COE-DuanXJ-1).

## Author Contributions

XD and XF conceived and designed the experiments. XF, YN, PW, ZZ, XL, ZX, ZQY, YZ, and QL fabricated the electrodes and performed the electrochemical characterization. XF, GL, BZ, and TJ performed the MRI studies. XF, JX, and TL conducted the insulation stability improvement research. XF, ZMY, and AC did the mechanical characterization. XD and XF wrote the manuscript. All authors discussed the results and commented on the manuscript.

## Conflict of Interest

The authors declare that the research was conducted in the absence of any commercial or financial relationships that could be construed as a potential conflict of interest.

## Publisher’s Note

All claims expressed in this article are solely those of the authors and do not necessarily represent those of their affiliated organizations, or those of the publisher, the editors and the reviewers. Any product that may be evaluated in this article, or claim that may be made by its manufacturer, is not guaranteed or endorsed by the publisher.
