# Supplementary material for: Carbon-Based Fiber Materials as Implantable Depth Neural Electrodes
Source: Front Neurosci. 2021 Dec 22;15:771980. doi: 10.3389/fnins.2021.771980 (PMC8730365; doi:10.3389/fnins.2021.771980)
Supplement: Supplementary file 1 [file Data_Sheet_1.pdf]

*Supplementary Information for*

# Carbon-based fiber materials as implantable depth neural electrodes

# Supplementary methods

## Electrode fabrication

The array CNT fibers (aCNTFs) were spun out of spinnable CNT arrays, which were grown from chemical vapor deposition (CVD) method as described previously (Jia et al., 2011). Briefly, CNT arrays were grown on Si substrates which were coated with 1-2 nm Fe film as catalysts. The growth was carried out at a temperature of 750 °C with $C_2H_4$ of 300 sccm as the carbon source diluted in $H_2$ of 200 sccm and Ar of 1000 sccm at the atmospheric pressure for 15 min. This gave vertically aligned CNT arrays consisting of double- and triple-walled CNTs with an average diameter of ~6 nm. The fibers were spun with a home-made spinning machine. CNT strips with a fixed width were drawn and then spun into fibers under draw and twist as reported before (Jia et al., 2011). During the spinning, a drop of ethanol is applied at the end of the triangular CNT strip to densify the CNTs.

Floating catalyst CNT fibers (fCNTFs) were obtained by twisting the continuous CNT yarns which were directly drawn from a vertical floating catalyst CVD system (Zhou et al., 2021). Ethanol solution containing 0.23 to 2.3 wt % ferrocene and 1.0 to 4.0 wt % thiophene was injected into the top end of the furnace tube at a rate of 0.08 to 0.25 mL/min, together with hydrogen as carrier gas at 400 to 800 sccm, for the floating catalyst CVD process. Continuous CNT assemble was formed at 1100°C to 1300°C in the furnace, blown out of the other end of the furnace tube by carrier gas, densified into CNT yarn by water and finally spun into fCNTFs by the same spinning method as the aCNTFs. The fCNTFs contained multi-walled CNTs with an average diameter of ~10 nm. The percentage of iron in the as-prepared fCNTFs was around 8 wt% as measured by thermogravimetric analysis (TGA, Supplementary Figure S4). The percentage of iron in the as-prepared aCNTFs was below the detection limit of TGA.

The graphene fibers (GFs) were prepared through a dimension-confined hydrothermal process using suspensions of graphene oxide (GO) (monolayer; thickness, 0.8–1.2 nm; sheet diameter, 0.5–5 μm)(XF002-2, Nanjing XFNANO Materials Technology, China)(Dong et al., 2012). In a typical preparation, an 8 mg/mL aqueous GO suspension was injected into a glass pipeline with a 0.5 mm inner diameter using a syringe. After being baked at 230 °C for 2 h with the two ends of the pipeline sealed, a GF matching the pipe geometry was produced. This preformed GF was then released from the pipeline by flow of $N_2$ and dried in air. The dried GF had a reduction in diameter to 30 μm. The electrical conductivity of aCNTFs, fCNTFs, GFs , and CFs was ~ $5.0\times10^4$ S/m, ~$3.4\times10^5$ S/m, ~$6.1\times10^2$ S/m, and ~$3.5\times10^4$ S/m respectively.

# Supplementary figures

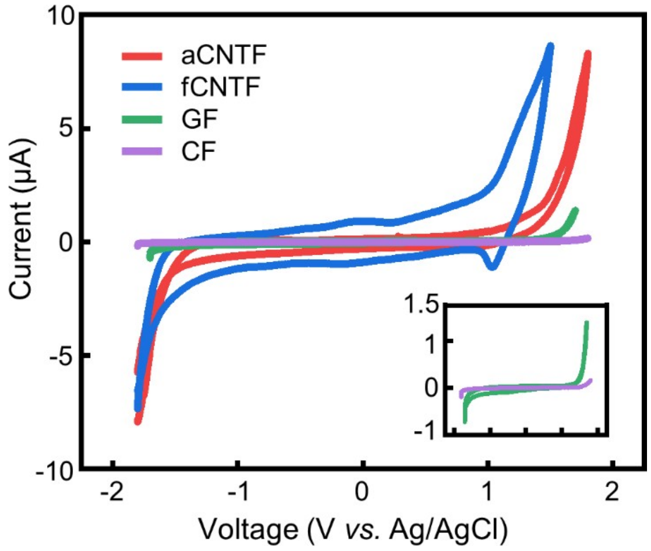


**Supplementary Figure S1.** Cyclic voltammogram for water window determination. The water oxidation and reduction potentials (water window) were determined as the potentials where steep increase in the current occurs. Inset, amplified data with x- and y-axis defined same as the main figure.

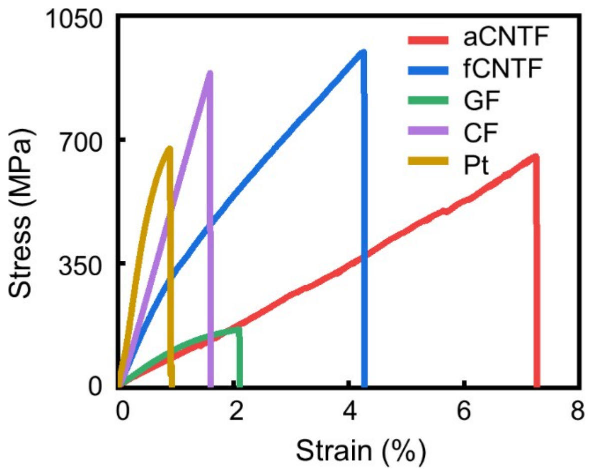


**Supplementary Figure S2.** Mechanical characterization. The stress-strain curves of different carbon-based fibers measured on a single-column testing instrument (Instron 5843, Instron Corp., USA).

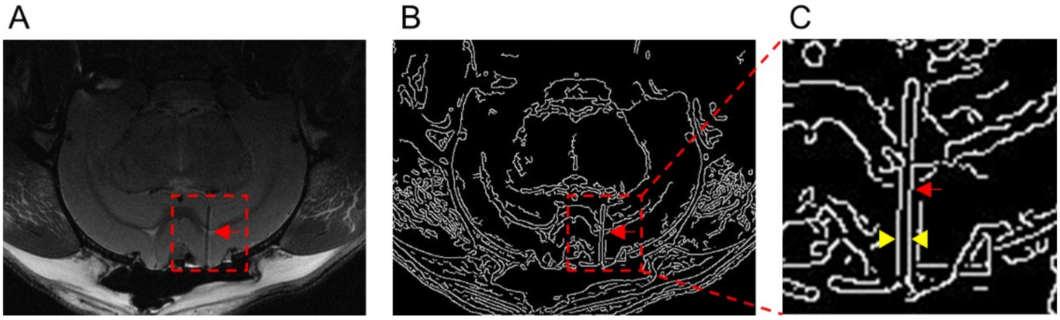


**Supplementary Figure S3.** MRI artifact measurement. (A) An original MRI image used for artifact measurement. (B) The generated image after edge detection using Canny Edge Detector using Matlab (R2018b, Mathworks, USA). (C) The enlarged image of the dashed box in B. The artifact and the detected artifact edge are pointed by the red arrows. The size of the artifact (marked by the yellow arrows) is measured directly from the detected edge.

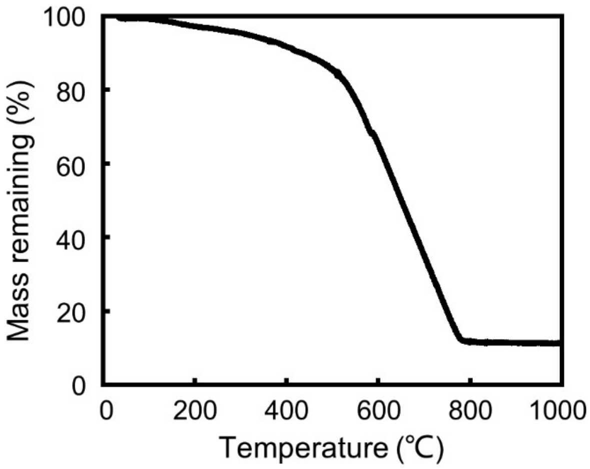


**Supplementary Figure S4.** TGA result of fCNTFs. The TGA was performed with the DSC-TGA thermal analyzer system (Labsys Evo, Setaram, France) in air atmosphere at a heating rate of 10 °C/min. The Fe was oxidized to $Fe_2O_3$ which accounts for ~11.5% of the whole weight. From the weight fraction of Fe in $Fe_2O_3$ of 70%, this gave weight fraction of Fe in fCNTFs as ~8%.

**Table S1.** Comparison on the electrochemical performance of carbon-based fiber electrodes with the published data.

| Materials | Active site area ($\mu m^2$) | Specific Impedance @1 kHz ($M\Omega \cdot \mu m^2$) | $CSC_c$ ($mC/cm^2$) | CIL ($mC/cm^2$) | Ref. |
|---|---|---|---|---|---|
| CNTF | 1450 | 20.4 | 372 | 6.52 (-1 V)* | Vitale et al., 2015 |
| CNTF | 314 | 87.9 | 278.21 | 3.52 (-0.6 V)* | Lu et al., 2019 |
| CNTF | 1962 | 5.9 | 2163 | 15.09 (-0.9 V)* | Alvarez et al., 2020 |
| aCNTF | 706 | 91.5 | 223.86 | 9.43 (-1.5 V)* | This work |
| fCNTF | 706 | 114.8 | 156.21 | 3.74 (-1.5 V)* | This work |
| GF | \ | \ | \ | 14, 19, 46, 62# | Apollo et al., 2015 |
| GF | 4416 | 66.7 | 889.8 | 10.10 (-1.5 V)* | Zhao et al., 2020 |
| GF | 169 | 1.9 | 946 | 10.34 (-0.9 V)* | Wang et al., 2019 |
| GF | 706 | 35.64 | 832.03 | 9.96 (-1.5 V)* | This work |
| CF | 36 | 246.2 | 3.01 | \ | Patel et al., 2015 |
| CF | 2500 | 2500 | 0.10 | \ | Gillis et al., 2018 |
| CF | 2238 | 298.6 | \ | 0.02 (-0.6 V)* | Hejazi et al., 2020 |
| CF | 706 | 1490.72 | 0.91 | 0.12 (-1.5 V)* | This work |

*: water reduction limit used for CIL determination.
\: not available.
#: calculated values.

## References


Alvarez, N. T., Buschbeck, E., Miller, S., Le, A. D., Gupta, V. K., Ruhunage, C., et al. (2020). Carbon Nanotube Fibers for Neural Recording and Stimulation. *ACS Appl. Bio Mater*. 3, 6478–6487. doi:10.1021/acsabm.0c00861.

Apollo, N. V., Maturana, M. I., Tong, W., Nayagam, D. A. X., Shivdasani, M. N., Foroughi, J., et al. (2015). Soft, Flexible Freestanding Neural Stimulation and Recording Electrodes Fabricated from Reduced Graphene Oxide. *Adv. Funct. Mater*. 25, 3551–3559. doi:10.1002/adfm.201500110.

Dong, Z., Jiang, C., Cheng, H., Zhao, Y., Shi, G., Jiang, L., et al. (2012). Facile fabrication of light, flexible and multifunctional graphene fibers. *Adv. Mater.* 24, 1856–1861.

doi:10.1002/adma.201200170.

Gillis, W. F., Lissandrello, C. A., Shen, J., Pearre, B. W., Mertiri, A., Deku, F., et al. (2018). Carbon fiber on polyimide ultra-microelectrodes. *J. Neural Eng*. 15, 16010. doi:10.1088/1741-2552/aa8c88.

Hejazi, M. A., Tong, W., Stacey, A., Sun, S. H., Yunzab, M., Almasi, A., et al. (2020). High Fidelity Bidirectional Neural Interfacing with Carbon Fiber Microelectrodes Coated with Boron‐Doped Carbon Nanowalls: An Acute Study. *Adv. Funct. Mater*. 30, 2006101. doi:10.1002/adfm.202006101.

Jia, J., Zhao, J., Xu, G., Di, J., Yong, Z., Tao, Y., et al. (2011). A comparison of the mechanical properties of fibers spun from different carbon nanotubes. *Carbon N. Y.* 49, 1333–1339. doi:10.1016/j.carbon.2010.11.054.

Lu, L., Fu, X., Liew, Y., Zhang, Y., Zhao, S., Xu, Z., et al. (2019). Soft and MRI compatible neural electrodes from carbon nanotube fibers. *Nano Lett*. 19, 1577–1586. doi:10.1021/acs.nanolett.8b04456.

Patel, P. R., Na, K., Zhang, H., Kozai, T. D. Y., Kotov, N. A., Yoon, E., et al. (2015). Insertion of linear 8.4 μm diameter 16 channel carbon fiber electrode arrays for single unit recordings. *J. Neural Eng*. 12, 46009. doi:10.1088/1741-2560/12/4/046009.

Vitale, F., Summerson, S. R., Aazhang, B., Kemere, C., and Pasquali, M. (2015). Neural stimulation and recording with bidirectional, soft carbon nanotube fiber microelectrodes. *ACS Nano*. 9, 4465–4474. doi:10.1021/acsnano.5b01060.

Wang, K., Frewin, C. L., Esrafilzadeh, D., Yu, C., Wang, C., Pancrazio, J. J., et al. (2019). High‐performance graphene‐fiber‐based neural recording microelectrodes. *Adv. Mater*. 31, 1805867. doi:10.1002/adma.201805867.

Zhao, S., Li, G., Tong, C., Chen, W., Wang, P., Dai, J., et al. (2020). Full activation pattern mapping by simultaneous deep brain stimulation and fMRI with graphene fiber electrodes. *Nat. Commun*. 11, 1–12. doi:10.1038/s41467-020-15570-9.

Zhou, T., Niu, Y., Li, Z., Li, H., Yong, Z., Wu, K., et al. (2021). The synergetic relationship between the length and orientation of carbon nanotubes in direct spinning of high-strength carbon nanotube fibers. *Mater. Des.* 203, 109557. doi:10.1016/j.matdes.2021.109557.
